# Dynamic assessment of listening effort by EEG alpha oscillations during an adaptive speech-in-noise test

**DOI:** 10.1038/s41598-025-17451-x

**Published:** 2025-10-02

**Authors:** Davide Simeone, Edoardo Maria Polo, Riccardo Barbieri, Alessia Paglialonga

**Affiliations:** 1 Cnr-Istituto di Elettronica e di Ingegneria dell’Informazione e delle Telecomunicazioni (CNR-IEIIT), Piazza Leonardo da Vinci, 32, 20133 Milan, Italy; 2 Politecnico di Milano, Piazza Leonardo da Vinci, 32, 20133 Milan, Italy

**Keywords:** Listening effort, Electroencephalogram, Speech-in-noise, Task demand, Auditory system, Cognitive neuroscience, Biomedical engineering

## Abstract

The task of speech recognition in noisy environments can be cognitively demanding for both hearing-impaired and normal-hearing individuals. Recent research emphasized brain oscillations, particularly alpha rhythms (8-13 Hz) from the electroencephalogram (EEG), as potential markers of processing auditory information in challenging listening contexts. However, most studies examined fixed listening demand with meaningful speech. Our study explores how alpha rhythms may quantify listening effort during an adaptive speech-in-noise task with reduced semantic complexity. For analysis, 11 participants with normal hearing were selected, and the individual speech recognition threshold was used to define two conditions with different task demands. Within each condition, multiple auditory stimuli were presented at different signal-to-noise ratios. The difference in listening demand between these conditions was reflected by reaction time and speech recognition performance. A cluster-based permutation test on EEG time-frequency data identified a fronto-central cluster in the alpha band. In-depth analysis of EEG dynamics revealed a significant difference in post-stimulus alpha event-related synchronization in a fronto-central cluster and a near-significant difference at central electrodes, providing preliminary support to the inhibition hypothesis. These findings further validate EEG-derived measures towards an accurate quantitative assessment of short-term neural responses in the context of a simple adaptive speech-in-noise test.

## Introduction

Listening effort (LE) is defined as the necessary recruitment of cognitive resources to complete a listening task, which means detecting semantic meaning in messages that could be subject to interferences such as noise, semantic complexity, or auditory impairment^1^. These kinds of challenges may translate into obstacles for everyday conversational engagement, and the resources constantly recruited to overcome LE conditions contribute, in turn, to the listening demand experienced during daily life listening tasks. A sustained increase in listening demand can lead to stress and fatigue, influencing individual performance and potentially leading to social withdrawal and low quality of life^1–3^. Although the problem of LE and its associated consequences are well recognized, many current approaches rely on subjective self-reported measures obtained through questionnaires related to the individual who experienced difficulties in everyday life, for example in relation to noise and social interactions^4^. However, a growing body of research is focusing on the study of LE-related information extracted from physiological measures. This shift reflects a broader interest in understanding how task-related demands modulate task demand in effortful listening. Since LE can be viewed as a specific manifestation of listening demand in auditory contexts, these physiological measures–particularly neural and behavioral responses–offer a valuable means to examine how listeners dynamically allocate cognitive resources in response to varying auditory demands. These measures hold great promise for estimating objective peripheral and central indices of listening demand and can be helpful not only to support clinical decision-making but also to improve our understanding of the underlying mechanisms associated with individuals’ ability to allocate resources to a listening task. Integrating physiological data into a comprehensive evaluation of human auditory perception in challenging listening situations could provide valuable information to improve hearing devices, such as hearing aids^5^, and customize human-machine interfaces. The literature on the quantification of cognitive processing load in response to auditory stimuli - which is often closely related to LE - through physiological measures is primarily focused on pupillometry^6^, although cardiovascular measures such as pulse ejection period and heart rate variability metrics are gaining ground in assessing motivation related to auditory tasks^7^. Furthermore, more direct approaches to detect brain activity include electroencephalogram (EEG) and brain imaging, e.g. using functional Magnetic Resonance Imaging (fMRI)^8^.

In the context of EEG, the body of research work is expanding, focusing on oscillatory dynamics observed during speech perception in noise. For instance, in an earlier study monosyllabic, bisyllabic, and trisyllabic German nouns were degraded in both their temporal envelope and spectrum and presented to 24 normal-hearing participants^9^. EEG data and subjective ratings on stimuli comprehensibility were collected. A monotonic relationship was found between the level of spectral acoustic detail in the speech signal and late alpha suppression in posterior-central channels, with the magnitude of suppression also correlating with comprehension ratings. As reported by Dimitrijevic and colleagues^10^, 14 normal-hearing young adults were exposed to digits at their speech recognition threshold (SRT) aiming for 50% intelligibility in two different listening conditions: passive and attentive. Alpha oscillations were observed only during attentive listening, with event-related synchronization in central/parietal sources evident when data were grand-averaged across participants, indicating a condition-dependent modulation of alpha activity. A similar study^11^ investigated 10 adult cochlear implant users under the same conditions. The results reported an increase in alpha power during digit presentation in the attentive condition, as well as a positive correlation between self-reported listening effort and alpha activity in the left inferior frontal cortex. In a study considering 29 healthy older adults^12^, ranging from normal to moderately impaired hearing, participants were presented with 2, 4, or 6 spoken digits amidst three different levels of background noise, corresponding to varying memory loads (2, 4, or 6 digits) and noise levels (-4, 0, and +4 dB with respect to SRT detected at 80% intelligibility). As hearing sensitivity progresses from normal to mild hearing loss, EEG alpha power from electrodes at the centro-parietal region was found to increase at low and moderate levels of memory load and noise. However, alpha power decreases as hearing loss progressed from mild to moderate degree for the highest levels of noise (-4 dB) and memory load (6 digits), suggesting the limitations of adaptive mechanisms in challenging auditory conditions. Moreover, McMahon and colleagues^13^ recorded EEG from 16 young adults while they performed a sentence recognition task involving speech, with either 16-channel or 6-channel vocoded noise, presented at various noise levels ranging from -7 to +7 dB Signal-to-Noise Ratio (SNR). The results revealed a significant interaction between channel vocoding and parietal alpha power, with the latter decreasing as SNR increased for the speech with more spectral detail. In summary, the literature provides evidence that EEG alpha activity reliably reflects LE and task demand during speech perception in noise. Alpha power tends to increase with moderate cognitive or acoustic load, indicating greater engagement, but it decreases when intelligibility is high or when demand exceeds cognitive capacity. The sensitivity of alpha activity to attention and task conditions supports its use as a neural marker of auditory effort and cognitive resource allocation.

Despite the cognitive processes involved in speech perception remaining unclear and being primarily explained through the lens of cognitive resources like working memory and selective attention, previous works like the ones outlined above have established the importance of neural oscillations, especially EEG alpha rhythms, in auditory processing in challenging listening conditions. These oscillations, commonly associated with the visual and somatosensory domains, are thought to play an important role in the context of auditory tasks, through the enhancement of task-relevant areas or the inhibition of task-irrelevant information regions expressed, respectively, as an increase or decrease of alpha power^14^. Overall, the involvement of specific brain regions in response to speech degradation has been extensively studied. However, the predominant focus on semantically meaningful auditory stimuli limits our understanding of how cognitive processing adapts to varying levels of listening demand induced by acoustic interference (e.g., background noise) in the context of speech. In addition, most studies examine fixed levels of task demand, failing to reflect the variability that characterizes real-life listening situations and differences in individual auditory abilities.

Building on the current understanding of cortical responses to auditory stimuli in challenging conditions, the current study investigates how neural rhythms can reflect the listening demand required during an adaptive speech-in-noise task where semantic complexity is minimized. The key questions driving this work are: (i) whether neural oscillations, particularly in the alpha frequency range, can serve as reliable markers for detecting differences in effortful listening due to degraded auditory stimuli across a range of SNRs; and (ii) how reducing semantic complexity influences the topographical response of alpha power, providing deeper insights into how cognitive factors shape the neural correlates of listening demand. Specifically, in this study an adaptive, multiple choice, self-administered speech-in-noise test (SNT) using meaningless intervocalic consonants is used and two groups of trials, presented at SNR levels higher than or lower than each subject’s SRT, are defined as conditions characterized by different task demand, respectively. These two groups of trials are analyzed and compared to observe possible neural and behavioral differences across them. Each condition includes auditory stimuli presented at different SNR levels. This approach offers a nuanced view of how individual differences influence neural responses to degraded auditory stimuli, providing insights that are more reflective of real-world listening conditions. Behavioural measures (i.e. reaction time and performance) were also analyzed as a function of task demand. A multivariate analysis including signals reflecting cardiovascular, autonomic, and brain responses was recently published by our team^15^. However, in our earlier study the neural responses were only analyzed in relatively long time windows and no significant differences in overall power spectral density (PSD) in the theta (4-7 Hz), alpha (8-12 Hz), and beta (13-30 Hz) frequency bands were observed. To gain a deeper understanding of the effects of task demand on EEG responses, this study introduces a new approach by applying a trial-by-trial time-frequency analysis on the EEG dataset recorded in our earlier study^15^ to analyze fast oscillatory dynamics in response to single, short, auditory stimuli.

## Methods

The examined EEG data were recorded in the context of an experiment in which a validated adaptive SNT^16,17^ was administered to normal hearing subjects while different physiological measures were also recorded (i.e., electrocardiography, blood volume pulse, respiration, pupil diameter^15^). All methods were carried out in accordance with relevant guidelines and regulations and the protocol was approved by the Politecnico di Milano Research Ethics Committee on July 14, 2021 (Opinion No. 29/2021). Written informed consent was obtained from all participants prior to the commencement of the study. Details about the study participants, SNT, EEG recording and analysis are presented in the followings.

### Participants

Twenty-one healthy adults (13 female, 8 male; age: mean=26.2 years, SD=1.5) were recruited. All participants were native Italian speakers and had normal hearing thresholds in both ears, as tested by pure-tone audiometry (pure-tone averaged thresholds at 500, 1000, 2000 and 4000 Hz < 20 dB HL in each ear) measured using a clinical audiometer (Amplaid 177+, Amplifon) with TDH49 headphones. Participants were required to refrain from smoking and consuming coffee for at least two hours prior to the experiment to limit the potential effects of caffeine and nicotine on physiological signals.

### SNT administration and task demand classification

Participants performed a monaural SNT^16,17^ in front of a screen computer monitor wearing UXD CT887 earphones and interacting by using a mouse. The adaptive SNT used in this study employs a three-alternative forced-choice (3AFC) task in combination with 1-up-3-down, variable step size staircase. Specifically, the staircase starts at a relatively high SNR (+8 dB) to ensure low initial task demand in individuals with normal hearing and it modifies the SNR adaptively using a 1-up-3-down algorithm targeting the 79.4% SRT, i.e. the SNR is increased after one incorrect response and decreased after three correct responses, with a ratio between upward and downward steps lower than 1 to optimize convergence and efficiency, as explained in detail in previous works^16,18,19^. The individual SRT, defined as the SNR corresponding to 79.4% intelligibility, is computed as the average SNR of the midpoints of the last four ascending runs. Stimuli are presented in random sequence and belong to a corpus of 12 nonsense intervocalic consonants (VCV: Vowel-Consonant-Vowel stimuli). At each trial, the stimuli were presented as follows: 0.8 s of quiet, 0.2 s of noise, 0.6 s of VCV in noise, 0.5 s of noise, with the level of noise adaptively adjusted. Participants were instructed to adjust the volume of clean VCV stimuli using a slider before the test to ensure a comfortable listening level throughout the test.

For each subject, to classify stimuli in two levels of demand, two groups of auditory stimuli were extracted from the staircase track and then defined based on the SNR relative to the SRT (i.e., “overSRT” condition: SNR> SRT; “underSRT” condition: SNR $\le$ SRT) and based on the following criteria: (i) Each group must consist of the data acquired in at least ten consecutive trials within the same SNT staircase, selected based on whether their thresholds fall above or below the SRT. This minimum ensures a balance between the dynamic, oscillatory nature of the adaptive SNT procedure–which can make it difficult to isolate long sequences of similarly classified trials–and the need for a sufficient number of trials to reliably observe event-related frequency changes in the EEG; (ii) The number of trials in the overSRT and underSRT conditions must be equal to avoid possible bias caused by unequal number of trials; iii) Both groups should be brought as close as possible to the SRT in order to capture the perceptual dynamics occurring around this critical point.

Ten subjects out of twenty one did not meet the first requirement due to the limited number of trials at SNR $\le$ SRT, whereas the data from the remaining 11 participants were analyzed using a trial by trial time-frequency approach, as described in the followings.

### EEG recording and data processing

The EEG signal was recorded using a DSI 24 headset, equipped with 19 dry electrodes placed on the scalp according to the international 10-20 system, with a sampling rate of 300 Hz and a 16-bit A/D converter.

EEG data was first preprocessed using the EEGLAB toolbox^20^ (Mathworks, version 2025.0.0). The raw signal was first filtered with a bandpass filter (1–30 Hz). Following a visual inspection of the filtered time signals, channels with a standard deviation exceeding the 99.7th percentile of the distribution of standard deviations across all channels were excluded. This led to the removal of a maximum of two channels per subject, one from the parietal region (Pz) for all subjects and one from the frontal region (F8) for five subjects. Artifact subspace reconstruction was applied to attenuate for possible artifacts with large amplitude. Eyeblink and movement-related artifacts were removed by transforming the sensor data into independent component space data using Independent Component Analysis (ICA)^21^. Components were then discarded based on the following probabilities of belonging to specific categories: less than 5% for brain-labeled components, greater than 70% for components labeled as eye, muscle, heart, line, and channel noise, and greater than 90% for components labeled as ’other’^22^. On average, 2.6 ± 1.4 components (min: 1, max: 6) were removed per subject.

**Fig. 1:**
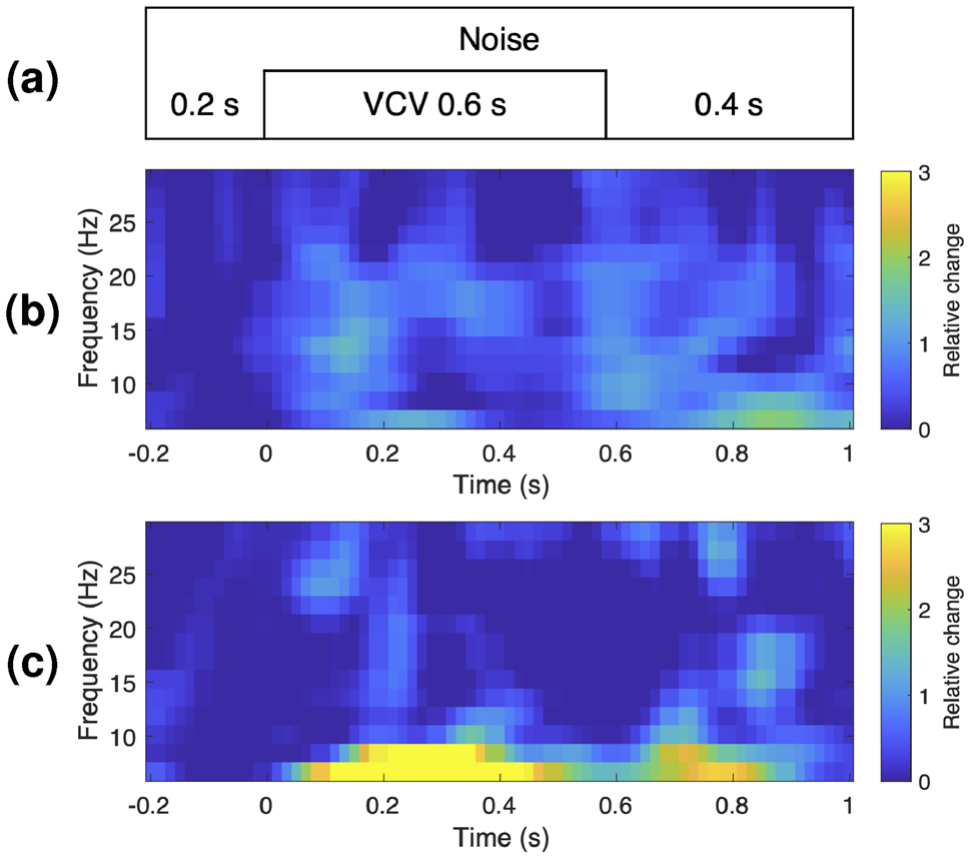
In (**a**) the structure of the auditory stimulus is illustrated. The first 200 ms are considered as reference for the RPCs computations, while t=0 indicates the beginning of the VCV recording. Figures (**b**) and (**c**) indicate the RPCs for the overSRT and the underSRT condition, respectively.

Data epoch segmentation and time-frequency analysis was performed using the Fieldtrip MATLAB toolbox^23^. The processed data was epoched between -1 and 2.5 s (noise onset: 0 s). Time-frequency representations were obtained through the application of complex Morlet wavelets, convolved with the signal in the frequency domain from -1 to 1 s in 20 ms steps and from 3 to 29 Hz with varying width going from 3 to 6. Relative power change (RPC) estimates were calculated for each time-frequency bin in the spectrogram by subtracting the baseline power (from a reference time window) from the power in the time-frequency bin, then dividing the result by the baseline power. Specifically, the baseline was defined as the time-averaged power from a pre-stimulus period (i.e., from -200 ms to 0 ms, which corresponds to presentation of noise only, with no VCV stimulus). An example obtained from a single electrode belonging to one of the subjects is illustrated in Fig. 1, where RPC differences are visible between the overSRT condition (Fig. 1b) and the underSRT condition (Fig. 1c), in particular in the theta and alpha bands.

The EEG responses epoched around each stimulus were averaged across all SNR levels within each SRT condition, overSRT and underSRT, to obtain an overall characterization of the neural response to speech-in-noise at low and high task demand, respectively. While changes in SNR are known to modulate neural responses, in this study our aim was to assess whether short-term EEG analysis can detect consistent neural patterns associated with suprathreshold and subthreshold speech-in-noise recognition tasks across a range of SNRs, rather than to isolate SNR-specific effects.

### Statistical methods

#### Cluster based analysis

For each channel, subject and condition a time-frequency representation was obtained averaging the RPCs across trials as in the example in Fig. 1, and used as input of a cluster-based permutation test^24^ to find possible spatio-temporal patterns showing significant differences between the two conditions. For each time-frequency-electrode bin, a paired t-test compares the distributions of values across subjects between underSRT and overSRT. Clusters are defined as groups of spatially and temporally adjacent time-frequency points where the RPCs differ significantly between conditions, as determined by these t-tests. For each cluster, a cluster-level statistic (T*) is computed as the sum of the test statistics (T) of the data points within that cluster. This procedure is applied to both the original data and several permutations ($2^{\text {n}}$, where n is the number of subjects) obtained by shuffling the labels that indicate the experimental condition , where the resulting T* values from the permutations form the null distribution. A cluster identified in the original data is considered statistically significant if its cluster-level p-value is lower than the significance threshold of a two-sided test, as applied to the null distribution.

The resulting clusters may be either positive or negative, depending on which condition is chosen as the reference. In this context, positive clusters indicate a significant difference where the condition of interest (underSRT) shows higher values compared to the reference condition (overSRT), whereas negative clusters indicate a significant difference where the reference condition shows higher values than the condition of interest.

The cluster-based permutation test is applied to all the time interval bins from 0.2 to 1 s, averaged over the theta and alpha bands, across all 19 channels. The test was implemented using the Fieldtrip toolbox in MATLAB^23^.

#### Statistical analysis of EEG markers and behavioural data

For each trial included in the underSRT and overSRT conditions, the following features were analysed: (i) SNR; (ii) dSRT, defined as the difference between the SNR and the subject’s SRT (iii) Reaction time, defined as the time from stimulus onset and individual response; (iv) Performance, defined as a binary variable (correct/incorrect answer), and (v) RPCs averaged across a time window of interest, frequency bands, and cortical areas that were identified as relevant.

Possible intra-individual differences in mean reaction time and mean RPCs between the two conditions were assessed using paired-sample t-tests. To account for inter-subject variability and repeated measures in subsequent trials, a linear mixed-effects (LME) model was employed to analyze the effects of condition, SNR, and dSRT (predictors) on RPC and reaction time. For the LME models, the RPCs were kept distinct for each trial. To ensure the normality of residuals from the fitted LME models, RPC and reaction time were shifted to be positive and subsequently log-transformed. Consequently, the fixed effect coefficient reflects the average change in the log-transformed response variable for a one-unit increase in the corresponding predictor variable. Applying an exponential function to the coefficient yields a transformed multiplicative factor, which indicates the average percentage change in the response variable for each unit change in the independent variable.

To model performance (defined as a binary outcome) through condition, SNR and dSRT, a generalized linear mixed-effects (GLME) model was used, assuming a binomial distribution for the response variable and applying a logit link function. In this model, the fixed effect coefficient represents the average change in the log odds of the response variable for a one-unit increase in the corresponding predictor variable. The application of an exponential function to the coefficient yields a multiplicative factor which indicates the average change in the odds (the ratio between the probability of correctly identifying an auditory stimulus and not recognizing it) for each unit change in the predictor variable.

Because participants may differ not only in their overall response levels but also in how they respond to specific conditions, we tested models with both random intercepts and random slopes. This allowed us to assess whether accounting for subject-specific variation in the effect of condition improved model fit. To determine the most appropriate model structure, we compared models using the Akaike Information Criterion (AIC) and the Bayesian Information Criterion (BIC). These metrics are especially useful when comparing models with different numbers of parameters—such as fixed versus random slopes—to assess whether added complexity improves model performance^25^. All statistical analyses were conducted using MATLAB^26^.

## Results

The dataset includes 11 subjects (7 female; age: 26.2±0.9 years; SRT: -16.2±1.6 dB) with 18±8 trials per condition (overSRT: 6.2±3.76 dB dSRT; underSRT: -2.6±0.9 dB dSRT). The mean trial duration was 3.10±0.65 s for the overSRT condition and 3.22±0.64 s for the underSRT condition. Data for each subject are illustrated in Table 1.

**Table 1:** Summary of the study dataset.

Subject ID	#trials/condition	Tested ear	Reaction time (s): Mean (S.D.)		Performance		SNR (dB): Mean (S.D.)	
			Over SRT	Under SRT	Over SRT	Under SRT	Over SRT	Under SRT
1	23	Right	2.7 (0.42)	2.8 (0.50)	1.0	0.91	-3.8 (6.32)	-19.4 (2.01)
2	14	Right	2.5 (0.17)	3.0 (0.71)	0.93	0.71	-11.4 (1.49)	-18.5 (1.70)
3	13	Left	2.6 (0.46)	2.4 (0.25)	0.85	0.85	-11.1 (1.35)	-16.0 (1.53)
4	24	Left	2.3 (0.27)	2.5 (0.40)	1.0	0.83	-4.2 (6.43)	-17.9 (1.72)
5	24	Right	2.5 (0.21)	2.9 (1.24)	1.0	0.83	-4.2 (6.43)	-17.0 (1.74)
6	14	Right	2.5 (0.24)	2.8 (0.56)	1.0	0.86	-13.5 (2.89)	-20.4 (1.69)
7	15	Left	2.5 (0.16)	2.6 (0.35)	0.93	0.87	-11.9 (3.75)	-17.8 (0.96)
8	14	Left	3.0 (0.48)	3.2 (0.74)	0.86	0.79	-14.7 (1.31)	-17.9 (1.02)
9	14	Left	3.0 (0.35)	3.1 (0.47)	1.0	0.86	-15.7 (2.28)	-22.4 (2.06)
10	12	Left	2.6 (0.32)	2.8 (0.69)	0.92	0.75	-11.2 (1.46)	-17.4 (0.58)
11	37	Right	2.9 (0.85)	3.3 (0.54)	0.97	0.81	-8.2 (7.61)	-20.7 (1.67)
**Grand Average (S.D.)**			**2.6 (0.23)**	**2.9 (0.30)**	**0.95 (0.057)**	**0.80 (0.057)**	**-10.0 (4.30)**	**-18.7 (1.81)**

For each participant, the number of trials per condition, the tested ear, and the mean and standard deviation (S.D.) of reaction time, performance, and SNR for each condition are shown. The last row displays the grand average and the between-subject standard deviation.

### Cluster based analysis

**Fig. 2:**
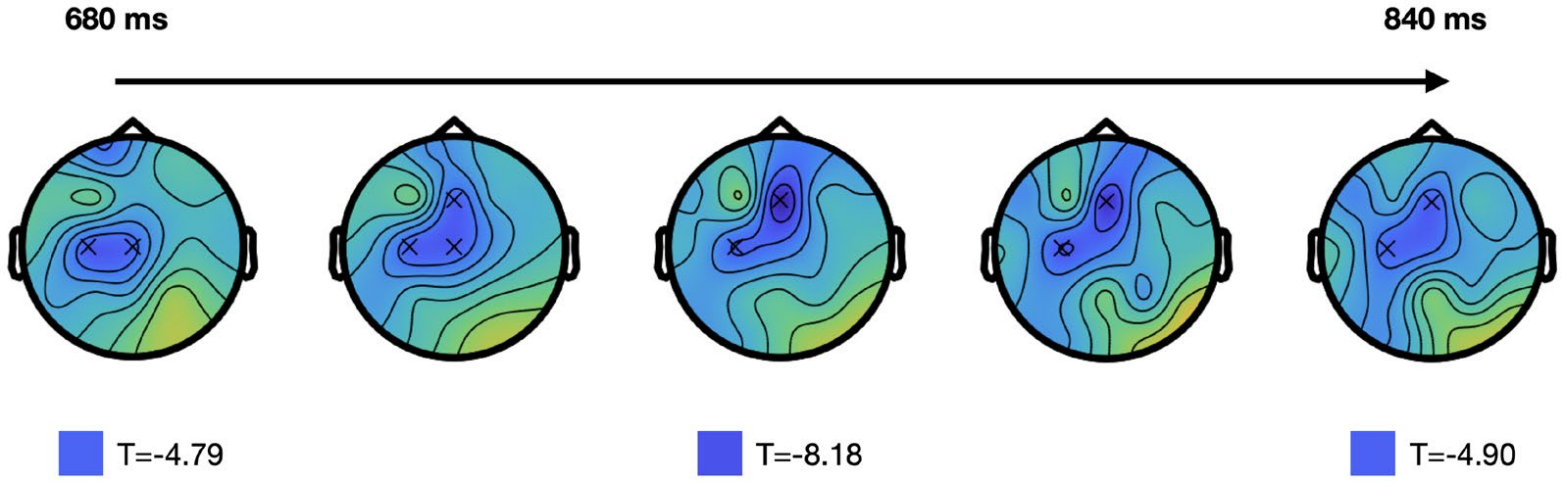
Temporal topographic pattern of the negative cluster identified by the cluster based permutation test applied on alpha. The cluster is composed by central and frontal electrodes (C3,Cz,Fz), with a varying pattern over a time window from 680 to 840 ms after stimulus presentation. The cluster level statistics reaches its absolute peak at 760 ms post VCV onset (T=-8.18).

The cluster-based permutation test revealed a statistically significant difference in RPCs between the overSRT and underSRT conditions in alpha, as evidenced by the presence of a negative cluster (T*=-55.13; $p < 0.01$) on the fronto-central channels. Specifically, the cluster-based permutation test indicated higher RPCs in the underSRT condition compared to the overSRT condition, whereas no significant difference was observed in the theta band. This statistically significant difference in RPCs in alpha was observed around 680 ms in the left-central cortical region, gradually extending toward the left parietal area and more prominently toward the midfrontal region, where it reaches its peak at 760 ms post-onset and then it attenuates until 840 ms (Fig. 2).

Based on the outcomes of the cluster-based permutation test, the following analyses were focused on the midfrontal, parietal and central regions. Both alpha and theta bands were considered, following evidence reported in the literature^27,28^.

### Time-varying dynamics of alpha and theta oscillations

**Fig. 3:**
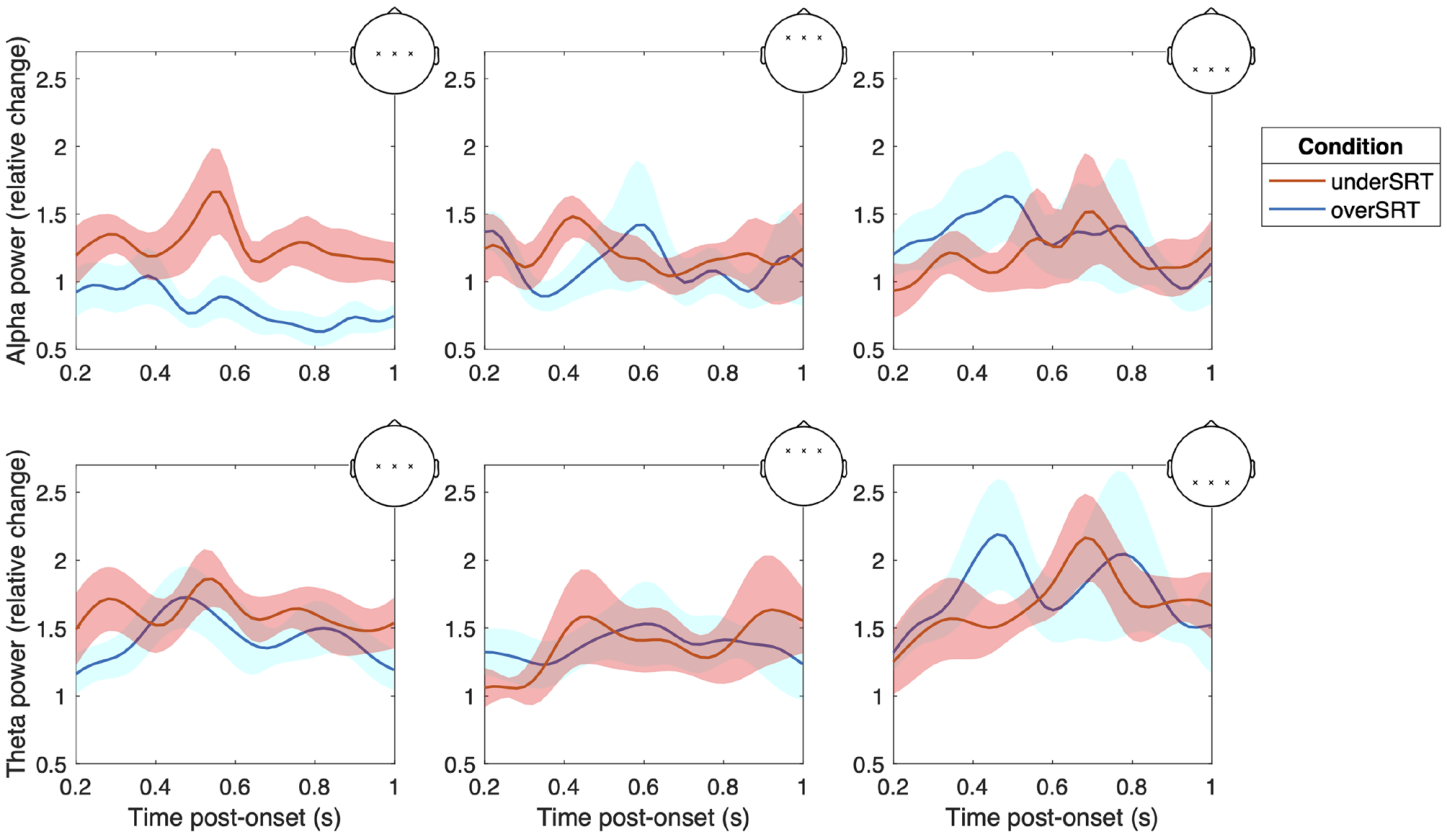
Grand-average RPCs for alpha (top panels) and theta (bottom panels) bands observed in the central (left-hand panels), midfrontal (center panels) and parietal electrodes (right-hand panels), for the overSRT condition (blue line) and underSRT condition (red line). The colored shaded areas correspond to ±1 standard error of the mean.

Figure 3 illustrates the grand-average RPCs in the alpha and theta bands for three different subsets of channels (in the midfrontal, central, and parietal areas). For the sake of clarity, the first 200 ms post-onset were discarded. Overall, most of the RPC trends shown in Fig. 3 exhibit largely overlapping patterns for the two conditions, except for the RPC trends observed in the central region in the alpha band. The difference in alpha RPC in the central region is evident both after the end of the stimulus (i.e., in the interval 0.6-1 s, in line with findings from the cluster-based test), and during the stimulus itself (i.e., in the interval 0.2-0.6 s). This qualitative difference was tested using statistical analysis, as detailed below.

### EEG statistical analyses

Following the previous qualitative analysis, single trial alpha RPCs for the central region (electrodes C3, Cz, C4) were averaged between 0.6 and 1 s post VCV onset to assess possible statistically significant differences during post stimulus auditory processing on a longer time window compared to that identified by the cluster based test (i.e., 0.68-0.84 s).

**Fig. 4:**
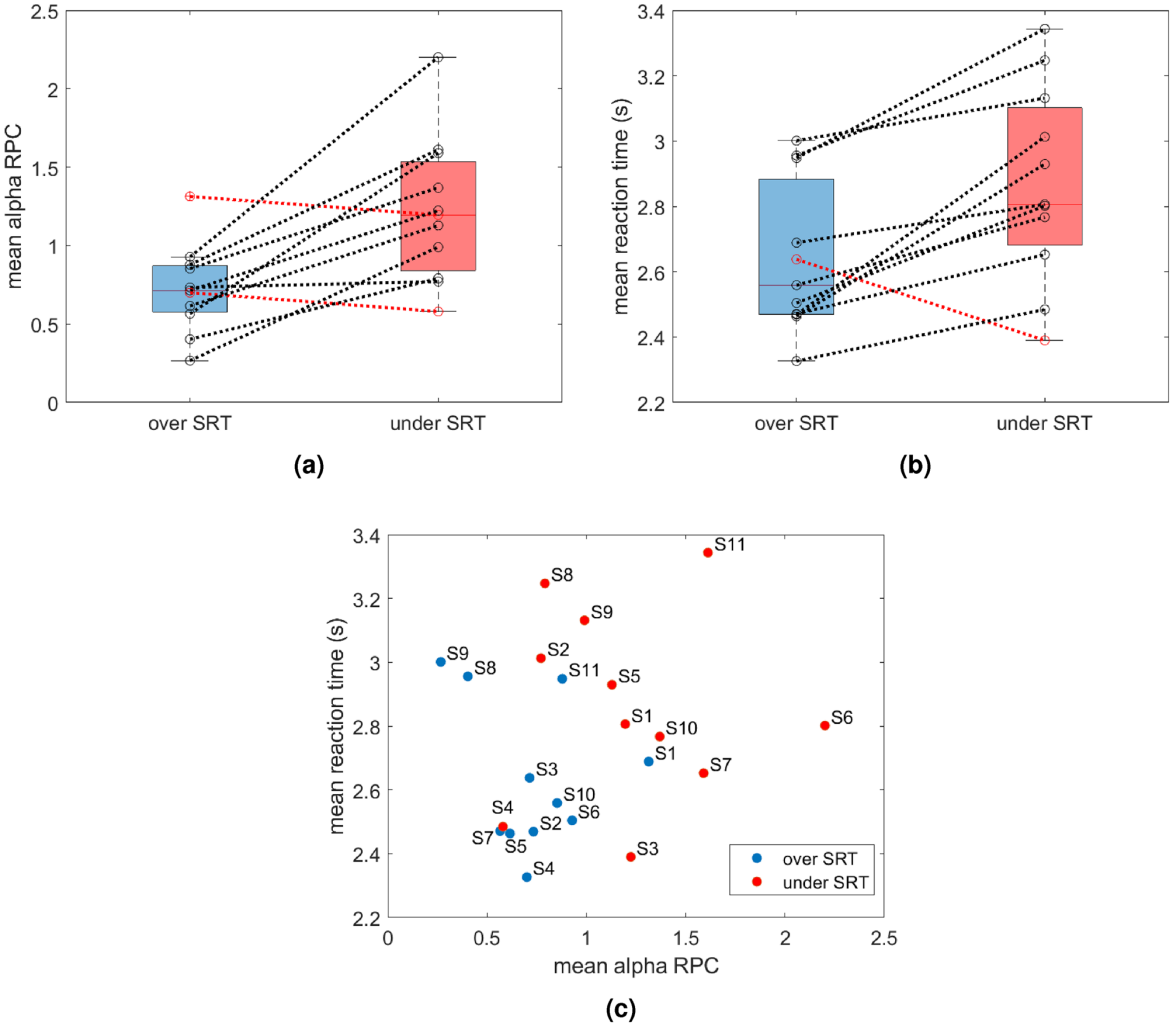
Distributions of subject average alpha RPC in the interval 0.6–1 s (top left) and average reaction time (top right) for the overSRT (blue boxplot) and underSRT (red boxplot) conditions. Red dotted lines connect instances in which the trend from overSRT to underSRT shows an opposite behavior compared to the overall trend. In the bottom center panel, both pieces of information are represented in a 2D scatter plot, with data points for both conditions labeled by subject ID.

The distributions of average RPCs across subjects are shown in Fig. 4a. Paired sample t-test indicated a significant difference in average RPC (p=0.004) between the two conditions in alpha, whereas no significant differences in average RPC in the theta band were observed (p=0.252) and therefore not shown. A total of 9 out of 11 subjects showed an average increase of RPC in alpha in the underSRT condition compared to the overSRT condition whereas only two subjects (i.e., subjects 1 and 4) showed an opposite pattern but the average differences were small (i.e., 0.11 and 0.12, respectively).

The results of the LME models used to assess the effect of different predictor variables (condition, SNR and dSRT) on single trial alpha RPC are shown in Table 2. From here onward, we will focus on the fixed effects models, as they provided a better fit (lower AIC and BIC values) when compared to the random slope models. For the fixed effect models, no significant effect was observed using SNR and dSRT as predictor variables, whereas the effect of condition (overSRT vs. underSRT) on alpha RPC was close to the significance level with a p-value of 0.051, indicating that the findings are suggestive but not conclusive regarding an effect of condition when the averaged data is considered. The fixed effect of condition as predictor variable was estimated as a log coefficient of 0.135. Conversion using an exponential function results in a linear value of 1.14, indicating an average 14% increase in the response variable (alpha RPC) from the overSRT to the underSRT condition. Applying the fixed-slope LME model using the same procedure described above, but selecting the cluster identified by the cluster-based permutation test (electrodes C3, Cz, Fz), would instead yield a significant effect (p = 0.017), indicating an average 18% increase in alpha RPC from the overSRT to the underSRT condition.

### Behavioural measures

Figure 4b shows that the reaction time was higher in the underSRT condition compared to the overSRT condition for most of the tested subjects, with the only exception being subject 3, who exhibited a similar distribution of percent correct in the two conditions (0.85±0.10), as shown in Table 1. An increased reaction time in the underSRT condition compared to the overSRT condition is consistent with the increased difficulty of the recognition task at lower SNR. Statistical analysis (paired samples t-test) indicated that the observed difference in average reaction times between the two conditions was significant (p=0.005). In line with these findings, the grand average performance was 95% in the overSRT condition and 80% in the underSRT condition (Table 1). Figure 4c illustrates, for each subject, the combined RPC and reaction time in each of the tested participants in the two conditions. The figure shows an overall inverse relationship between RPC and reaction time and a clear separation of data from the two conditions.

The results obtained by applying LME (outcome: reaction time) and GLME (outcome: performance) models are presented in Table 2. Fixed slope models proved in most cases to be a better fit for all response variables compared to random slope models. All predictor variables (condition, SNR, and dSRT) had a significant effect on reaction time and performance (p<0.001). In terms of condition, the models estimated an average effect consisting of 8.5% increase of reaction time and an average 80.3% decrease in the odds of success going from the overSRT to the underSRT condition. Furthermore, each unit increase in SNR resulted in a 0.79% decrease in reaction time and a 24.9% increase in the odds of success. Similarly, each unit increase in dSRT led to a 0.78% decrease in reaction time and a 30.5% increase in the odds of success.

**Table 2:** Results obtained from LME models (alpha RPC, reaction time) and GLME models (performance) using both the fixed slope and the random slope modes.

	Alpha RPC		Reaction time		Performance	
	Fixed slope	Random slope	Fixed slope	Random slope	Fixed slope	Random slope
Condition						
Slope (SE)	0.135 (0.069)	0.128 (0.075)	0.082 (0.017)	0.080 (0.018)	-1.624 (0.401)	-1.624 (0.406)
t-value	1.960	1.718	4.940	4.398	-4.003	-4.003
p-value	0.051	0.087	<0.001	<0.001	<0.001	<0.001
AIC	870.38	872.7	-269.17	-267.41	2230.7	2234.7
BIC	886.43	896.77	-253.12	-243.34	2242.7	2254.8
SNR						
Slope (SE)	-0.002 (0.005)	-0.002 (0.006)	-0.008 (0.001)	-0.007 (0.001)	0.222 (0.049)	0.202 (0.045)
t-value	-0.441	-0.283	-6.535	-5.840	4.568	4.478
p-value	0.660	0.777	<0.001	<0.001	<0.001	<0.001
AIC	874.01	875.12	-285.99	-284.17	2283.3	2281.8
BIC	890.06	899.18	-269.94	-260.1	2295.4	2301.8
dSRT						
Slope (SE)	-0.002 (0.005)	-0.002 (0.005)	-0.008 (0.001)	-0.007 (0.001)	0.266 (0.056)	0.266 (0.056)
t-value	-0.483	-0.362	-6.344	-5.712	4.758	4.758
p-value	0.630	0.717	<0.001	<0.001	<0.001	<0.001
AIC	873.97	876.08	-283.86	-282.00	2355.3	2359.3
BIC	890.02	900.15	-267.81	-257.93	2367.3	2379.4

The predictor variables are arranged in rows whereas the response variables are arranged in columns. As the data were transformed before applying the models, the coefficients are not expressed in their original scale.

## Discussion

The goal of this study is to demonstrate that it is possible to identify short-latency neural responses able to characterize the listening demand associated with speech-in-noise recognition as a function of task demand. To modulate task demand, the noise level was varied based on individual performance in an adaptive SNT^16^. This enabled both adjustment of the test difficulty based on the individual performance and personalized definition of two levels of task demand (i.e., underSRT and overSRT conditions), defined with respect to the individual SRT^15^.

Two main findings emerged from the analysis of EEG recordings measured in a group of young healthy participants with normal hearing. First, a spatiotemporal cluster in the fronto-central area was identified, associated with significant condition-related differences in cortical responses after the stimulus, as estimated using RPC of alpha power (Fig. 2). Second, a difference in alpha event-related synchronization between the two task demands was observed in the central region, supporting the inhibition theory and in line with the well-known role of alpha waves in suppressing task-irrelevant channels^29^ (Fig. 3, 4a).

The classification of task demand using the two conditions here defined was confirmed by the analysis of behavioural measures (Fig. 4b, Table 2). Reaction time, which is commonly correlated with cognitive effort^30^, has been shown to increase, at condition level, with higher task demands, while the probability of an incorrect response in the 3AFC task significantly increased. These results are in line with previous findings indicating that greater cognitive effort is required to associate the sublexical information represented by VCV auditory stimuli with the corresponding semantic representation that can be retrieved from long-term memory when a mismatch occurs between the two, leading to a slowdown in information processing^31^. At the single trial level, the definition of the dSRT metric allowed for a nuanced analysis of the trial’s position in relation to SRT, offering a more precise definition of individual task demand compared to the binary classification of trials as above or below the SRT. Statistical analysis (Table 2) also showed that dSRT has a stronger effect, compared to the SNR, on the probability of a correct response, suggesting that the ability of participants to accurately identify the target stimulus is more influenced by how far the stimulus is from the SRT than by the absolute SNR. Although SRTs were relatively similar across participants in this dataset, dSRT may offer greater insight in more heterogeneous populations, where individual differences in auditory abilities and SRT are more pronounced. This possibility should be carefully examined and controlled for in future studies.

The RPC difference between conditions identified through the cluster-based permutation test corresponded to a fronto-central cluster of three electrodes, with their activity varying over the time window from 680 to 840 ms after the VCV stimulus (post-onset). This latency can be interpreted as representative of slow alpha modulations, in line with previous findings^9^. Given that the tested ear is nearly evenly distributed between the tested subjects, the identified common cluster is likely to be related to a stage of processing not substantially impacted by the lateralization of neural responses to monaural stimuli. The identified cluster, due to its proximity to the premotor and prefrontal cortices, suggests a difference in high-level cortical speech processing, which commonly acts on speech degradation through neural activation^32^. The premotor and prefrontal regions are typically associated with networks involved in verbal memory and the allocation of cognitive resources, such as attention^8^. Although semantic complexity was minimized in the current task (e.g., due to use of nonsense VCV stimuli in multiple choice task), the activation of these high-level areas suggests that cognitive processes beyond basic acoustic features are still engaged, demonstrating an interaction with the informational content of the stimuli rather than solely the energetic content^33^.

The results of the cluster-based permutation analysis informed further analysis for investigating multiple cortical areas, specifically focusing on the role of oscillatory modulations in excitatory/inhibitory dynamics between the two conditions (Figure 3). An observable difference was detected in central alpha activity, with power changes similar to those observed in response to digits in the centro-parietal region in previous studies^12^. Although event-related synchronization may be explained by the role of alpha modulations in disengaging task-irrelevant auditory processing units^14^, the key difference observed in our result was a limited alpha enhancement in the low-demand (overSRT) condition, especially when the central region is compared to the frontal and parietal regions. While interpretations regarding cortical involvement must remain tentative without source localization, one speculative explanation is that the central region is actively involved in the facilitation of verbal memory processing when the auditory stimuli are more intelligible due to their low level of complexity. This could lead to a reduction of the cognitive load required to encode the relevant information and ignore energetic or informational masking, thereby minimizing the need for typical alpha inhibition seen in other cortical areas. In contrast, the frontal and parietal regions, which are primarily involved in semantic integration and attentional control, maintain their role in filtering out distractions from degraded speech stimuli across both low and high intelligibility conditions, but without noticeable differences. This lack of modulation could be attributed to the limited semantic complexity of the stimuli and the relatively small variation in attentional control engagement across intelligibility levels, which may not have been sufficient to elicit measurable differences in neural activity. Due to the spatial limitations of EEG, especially in the absence of source localization, these region-specific inferences should be interpreted with caution.

In this study, both inter- and intra-individual variability in brain responses were accounted for by analyzing the RPCs averaged during the interval following the offset of the auditory stimuli (0.6–1 s) (Fig. 4a). This was done using a paired t-test to assess within-subject differences, alongside the fitting of a linear mixed-effects (LME) model to capture both within- and between-subject variability (Table 2). When the RPCs were averaged across trials for each subject, within-subjects statistical analysis showed a significant difference in average RPCs between the two conditions, with most of the subjects showing an average increase in RPC with increased task demand (Fig. 4a). However, when data from each trial in each subject were used to fit the LME model, our analysis revealed a non-significant effect of condition, SNR, and dSRT on alpha power, although for condition only the p-value was close to the significance threshold. This relates to the application of the LME model to a region that only partially overlaps with the one identified by the cluster-based test–which would have shown a significant main effect of 18%, as the analysis was deliberately constrained to canonical cortical regions in order to facilitate anatomical interpretability and align with prior literature.

Overall, the findings of this study confirm the importance of assessing short-term neural responses in speech-in-noise recognition, taking into account inter-individual variability in speech recognition abilities and neural responses. However, the results should be carefully considered due to the reduced sample size, which may affect the reliability of the statistics and may not reflect the variability found in more diverse or representative samples, thus limiting the generalizability of the findings. Further investigation in a larger, more representative cohort of individuals is needed to reduce potential bias and further validate these findings, expanding their applicability. The reduced spatial resolution, due to the relatively low number of channels employed, is a relevant limitation of the ability to precisely investigate neural responses in different cortical areas. For instance, while there is evidence in the literature (32 EEG channels^13^; 128 EEG channels^12^) suggesting involvement of the parietal area in response to speech degradation^34^, no significant condition-related differences in activation in this area were detected in the current study. This may be explained by parietal activity being partially captured by central electrodes, due to the limited number of electrodes used. Additionally, a source localization analysis, which would require a greater amount of electrodes, will be valuable tool to gain more insight into the precise location of brain activity, moving beyond just the superficial response^35^. Another limitation of this study may be associated to the use of a dry EEG cap, which may have masked some of the responses due to a lower signal-to-noise ratio than what is typically obtained with gel-based systems. This, in turn, may have led to the potential discarding of meaningful neural information through the removal of entire channels or ICA components. While the use of a dry EEG allowed for a quicker and simpler setup, which is useful to improve comfort, it may have limited our ability to perform a nuanced analysis of the different roles of cortical areas in speech degradation processing compared to wet electrode systems which can achieve higher signal quality^36^. One more limitation arises from pooling data from subjects who received stimuli monaurally in either the left or right ear, which may have limited our ability to assess possible spatiotemporal effects associated with auditory processing that typically occurs in the temporal lobe contralateral to the tested ear^37^. Finally, the structure of the adaptive SNT protocol here used may have hidden specific patterns due to the subjects’ adaptation to progressively less intelligible stimuli, compared to alternative, longer fixed-levels protocols^38^. Future research is needed to address these limitations, for example by using a higher number of electrodes to enhance spatial resolution. Also, source localization and precise spatial information will be needed to correctly interpret the mechanisms underlying the observed findings. Moreover, analysis of brain responses in a larger number of participants will be necessary to assess possible laterality effects and develop more robust models and statistical analysis, ultimately supporting a more detailed understanding of the cortical involvement in speech degradation processing.

## Conclusions

The results of this study offer limited support to the inhibition framework, as they reveal alpha event-related synchronization despite increased variability in SNRs across conditions, suggesting that alpha activity may play a role in functionally inhibiting task-irrelevant or distracting auditory input. Notably, differences in alpha activity were most prominent over central EEG channels, particularly when participants listened to speech with reduced semantic content. This central involvement suggests that, in the absence of strong semantic cues, cognitive effort may be reflected more strongly in central rather than parietal regions–a finding that warrants further investigation. Additionally, the potential lateralization of neural responses depending on the ear of stimulation should be explored in more detail, which would require a larger sample size and increased spatial resolution to ensure sufficient statistical power and support a more precise interpretation of the underlying mechanisms. These findings demonstrate that a simple, adaptive speech-in-noise recognition task can effectively capture short-term neural dynamics related to task demand, offering a promising approach for assessing auditory cognitive processing. These findings might be further validated to support the development of smart, non invasive, personalized hearing assistance devices.

## Data Availability

The data that support the findings of this study are available upon request by contacting the corresponding author, D.S..
